# Reversible opacification of hydrophobic acrylic intraocular lens- two cases report

**DOI:** 10.1186/s12886-017-0509-0

**Published:** 2017-06-30

**Authors:** Dong Ju Kim, Roy S. Chuck, Jimmy K. Lee, Choul Yong Park

**Affiliations:** 1 Department of Ophthalmology, Dongguk University, Ilsan Hospital, 814, Siksadong, Ilsan-dong-gu, Goyang, Kyunggido 410-773 South Korea; 2 Department of Ophthalmology and Visual Sciences, Montefiore Medical Center, Albert Einstein College of Medicine, Bronx, NY USA

**Keywords:** Cataract, Intraocular lens, Hydrophobic, Acrylic, Reversible, TASS

## Abstract

**Background:**

The opacification of the intraocular lens (IOL) can cause significant visual deterioration. It is known that opacity of hydrophobic acrylic IOLs is rare. We report 2 cases of reversible optic opacification of hydrophobic acrylic intraocular lenses (Tecnis ZCB00, Abbott), observed within 2 months after uneventful cataract surgery.

**Case presentation:**

Case 1: Uneventful cataract surgery was performed on the left eye of an 86-year-old diabetic man with chronic open-angle glaucoma. A hydrophobic acrylic intraocular lens (IOL; Tecnis ZCB00, Abbott, Lake Bluff, IL) was implanted in the bag. Eye drops containing 0.5% levofloxacin and 1.0% prednisolone were used after surgery along with topical anti-glaucoma medications. At 7 weeks postoperative, cloudy, concentric IOL opacification developed, accompanied by decreased visual acuity and increased intraocular pressure. However, the opacification completely disappeared after 9 weeks.

Case 2: Uneventful cataract surgery was performed on the left eye of a 72-year-old woman. A hydrophobic acrylic IOL (Tecnis ZCB00) was implanted in the bag. At 2 weeks postoperative, cloudy, concentric IOL opacification developed, accompanied by ocular discomfort. After 4 weeks, opacification and discomfort completely disappeared.

**Conclusions:**

We observed two cases of completely reversible opacification of hydrophobic acrylic IOLs. The exact nature of the transient opacity remains unclear, but an inflammatory origin cannot be completely ruled out.

## Background

Although millions of cataract surgeries using posterior chamber lens implantation are performed worldwide each year, intraocular lens (IOL) opacification remains a serious complication that can affect visual acuity. The causes of IOL opacification are various and usually unclear. For this reason, the materials and design of IOLs have continuously improved. It is known that hydrophobic acrylic IOLs have a low incidence rate of posterior capsule opacification (PCO), discoloration and calcification compared to IOLs of different composition [1, 2]. One previous study reported that opacification of hydrophobic acrylic IOLs occurred when the IOLs were inserted in a piggyback manner [3]. The attachment of lens epithelial cells on IOL surface contributes to piggyback IOL opacification. Acrysof IOL (SA60AT, Alcon, TX, USA) showed more lens epithelial cell attachment compared to Tecnis IOLs (ZCB00, Abbott, Lake Bluff, IL) in an animal study [4]. Most IOL opacities are generally irreversible and eventually require clinical intervention for clearance.

Recently, we encountered two cases of IOL opacification that developed within 2 months after hydrophobic acrylic IOL implantation (Tecnis ZCB00). The white, semilucent opacification grew from the periphery to the center of the optic. After medical treatment, opacification disappeared from the center to the periphery of the IOL in both cases.

## Case presentation

### Case 1

**Fig. 1:**
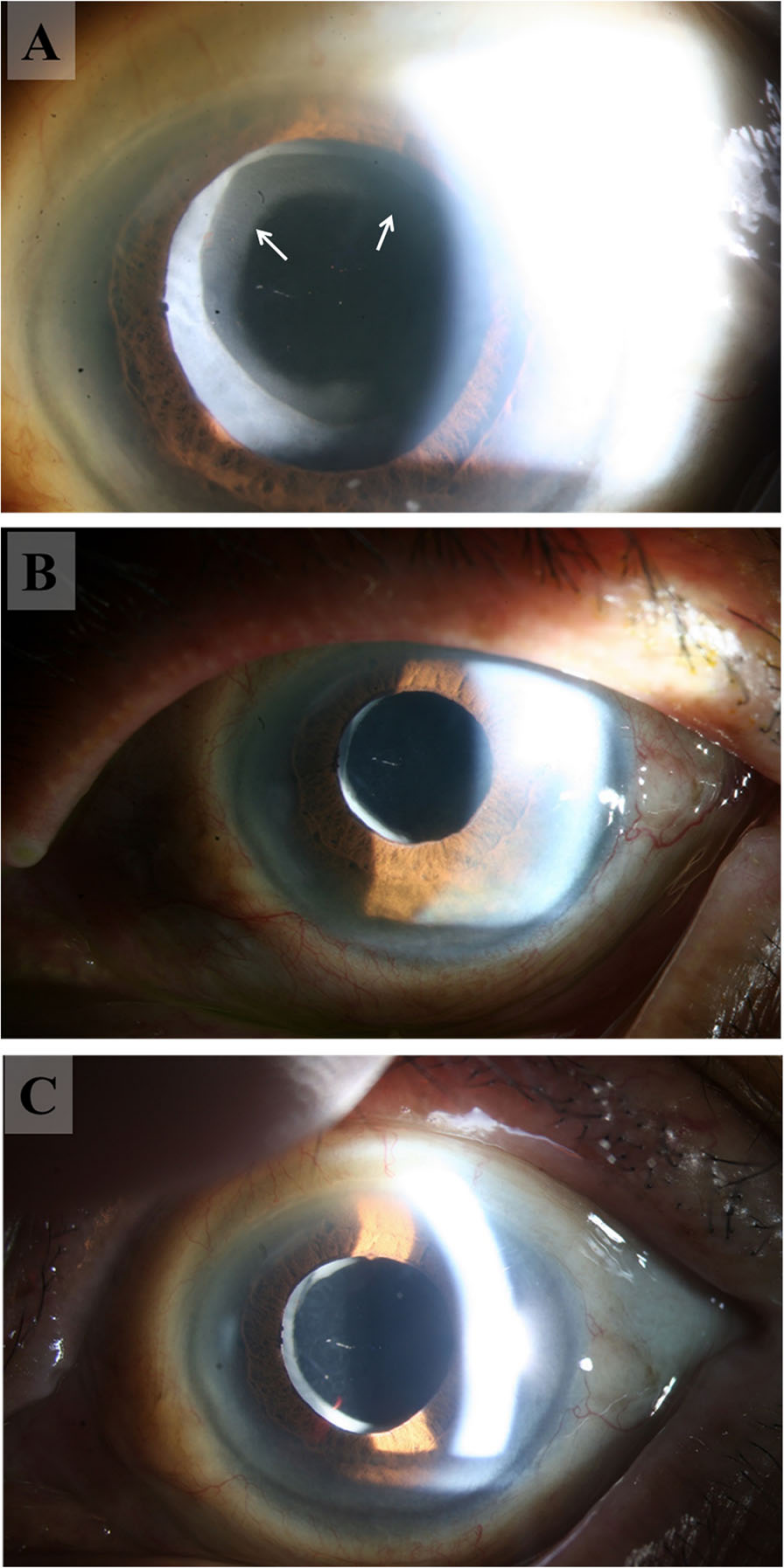
**a** Cloudy, concentric IOL opacity (*arrows*) with decreased visual acuity and increased IOP were developed 7 weeks after phacoemulsification and IOL implantation. Significant IOL opacity covered the anterior surface of the IOL, without a chamber reaction. The opacity was connected to the anterior capsulorhexis margin. **b** Five weeks after the first detection, IOL opacity had almost disappeared, and the patient had improved visual acuity and normalized IOP. **c** Another 4 weeks later, the IOL opacity was completely cleared

An 86-year-old Korean man was taking medication for hypertension (losartan potassium [Cozaar, MSD, Kenilworth] and lercanidipine hydrochloride [Zanidip, LG, Seoul]), diabetes mellitus (gliclazide [Diamicron, Servier, Neuilly sur Seine]), chronic obstructive pulmonary disease combined with asthma (budesonide [Pulmican, Kuhnil, Seoul], doxofylline [Asima, Bukwang, Seoul], erdosteine [Erdos, Daewoong, Seoul], and salbutamol sulfate [Ventolin, GSK, Brentford]), and benign prostatic hyperplasia (tamsulosin hydrochloride [Harnal-D, Astellas, Tokyo]). Primary open-angle glaucoma of both eyes was controlled with Cosopt (MSD, Kenilworth) and Xalatan (Pfizer, New York), keeping the intraocular pressure (IOP) between 13 and 15 mmHg. The patient had no history of uveitis. He had sequential phacoemulsification and hydrophobic acrylic IOL (Tecnis ZCB00) implantation in both eyes with 1 week between procedures. Surgery was performed uneventfully through a clear corneal incision, and a dispersive ophthalmic viscosurgical device (4% sodium chondroitin sulfate, 3% sodium hyaluronate [Viscoat, Alcon, Fort Worth]) was used. Preoperatively, the corrected distance visual acuity (CDVA) was 20/80 in each eye. One day after surgery, the CDVA had improved to 20/40 in the right eye and 20/50 in the left eye. Postoperative medication included 1.0%-prednisolone eyedrops (Pred-Forte, Allergan, Waco) and 0.5%-levofloxacin eyedrops (Cravit, Santen, Osaka), with one drop each, four times daily for 4 weeks. To eliminate the risk of pseudophakic cystoid macular edema, we discontinued both cosopt and xalatan for 4 weeks after the surgery. After 4 weeks, the anti-glaucoma medications that were used preoperatively were restarted. Seven weeks postoperatively, the patient was still using the remnants of the Cravit and Pred-Forte, and he complained of decreased visual acuity in the left eye; CDVA was 0.05 in the left eye. Significant opacification covered the anterior surface of the IOL without a chamber reaction. No evidence of inflammation was observed upon fundus examination. The angle was wide open upon gonioscopic examination, but IOP increased to 31 mmHg in the left eye. Cravit and Pred-Forte were discontinued, and Alphagan (Allergan, Waco) was added to the left eye. Five weeks later, IOL opacification had decreased, and the patient had improved visual acuity (20/100) and IOP (17 mmHg). Another 4 weeks later, the IOL opacification had almost disappeared from the left eye (Fig. 1). However, further follow-up examination was not possible because the patient died of aspiration pneumonia.

### Case 2

**Fig. 2:**
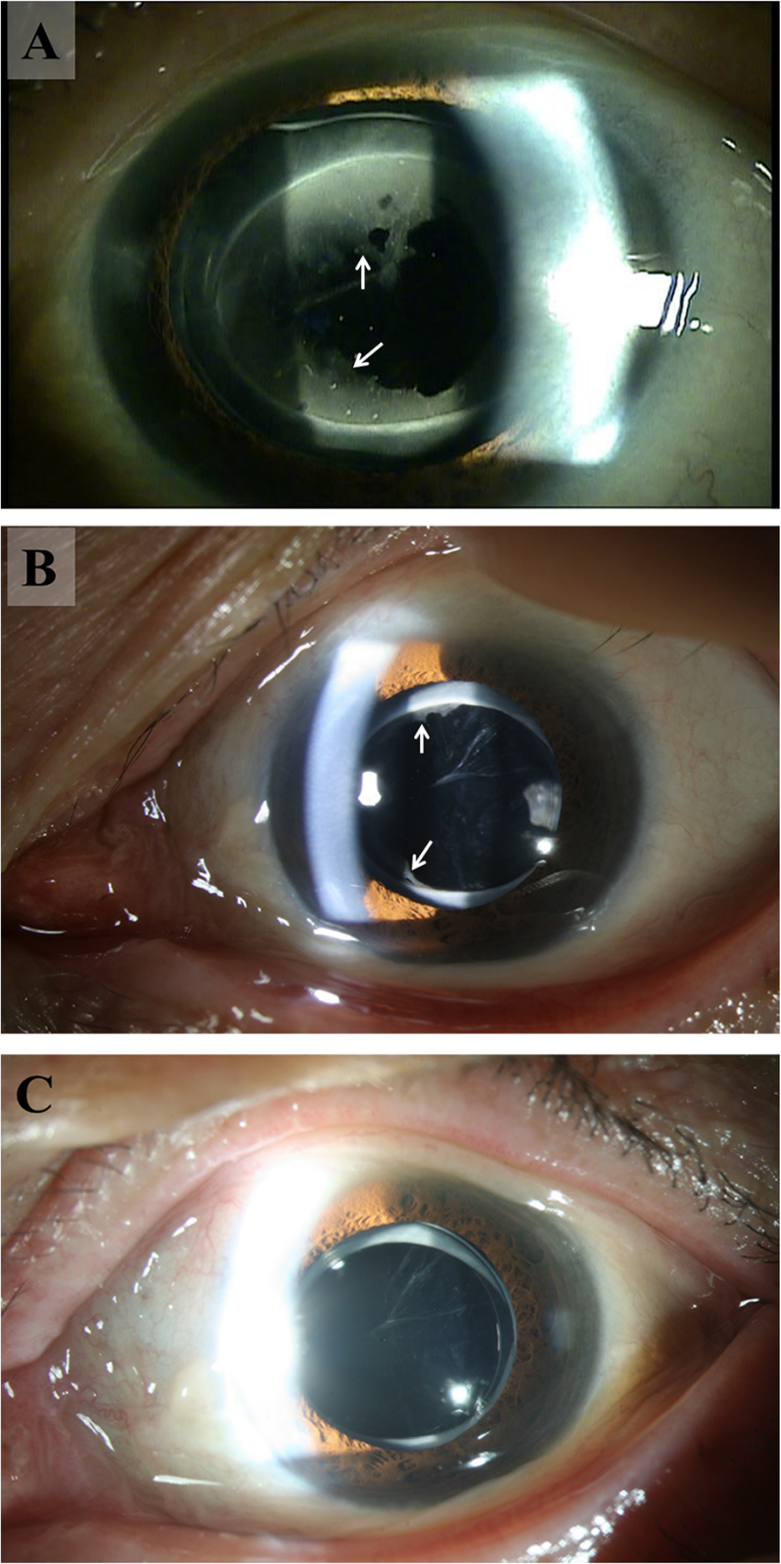
**a** Significant opacity (*arrows*) covered the anterior surface of the IOL, save the central area, accompanied by ocular discomfort at 2 weeks after cataract surgery. A very mild anterior chamber reaction was observed. **b** Two weeks after using a potent topical steroid, IOL opacity was significantly decreased, and resolution of the anterior chamber reaction was seen. **c** Another 2 weeks later, both IOL opacity and ocular discomfort had completely disappeared, and the CDVA was 20/20

A 72-year-old Korean woman was taking medication for angina (aspirin [Bayer, Leverkusen] and nitroglycerin, if needed). She had no history of uveitis and was diagnosed with a senile cataract. She underwent phacoemulsification and hydrophobic acrylic IOL (Tecnis ZCB00) implantation in the left eye. Surgery was performed uneventfully through a clear corneal incision, and a dispersive ophthalmic viscosurgical device (Viscoat) was used. Preoperatively, the CDVA was 20/25 but with blurred vision in the left eye. Postoperative medication included 0.1% fluorometholone (Flumetholone, Santen, Osaka) and 0.5%-moxifloxacin eyedrops (Vigamox, Alcon, Fort Worth), with one drop each, four times daily. Two weeks after surgery, the patient complained of discomfort in the left eye, but CDVA was still 20/25. Significant IOL opacification covered the entire anterior surface of the IOL except for the central area, with very mild anterior chamber reaction. The 0.1% Flumetholone was changed to 1.0% Pred-Forte, with one drop four times a day. Two weeks later, IOL opacification had decreased with the resolution of the anterior chamber reaction. Another two weeks later, both IOL opacification and ocular discomfort had completely disappeared, and the CDVA was 20/20 (Fig. 2).

## Discussion

Hydrophobic acrylic IOLs are increasingly being used because of higher biocompatibility and low incidence of PCO, compared to other IOLs [5, 6]. However, even hydrophobic acrylic IOLs have optical complications, known as glistening and light scattering. Glistening is the accumulation of fluid in the form of microvaculoes inside the IOLs, and light scattering on the surface of IOL is related to a hydration-related substance [3, 7–10]. Usually, these two types of opacifications inside the optics are known not to affect visual acuity and optical aberrations [8, 11]. Unlike the two irreversible complications above, the reversible opacification observed in our cases caused visual impairment and ocular discomfort.

The causes of IOL opacification are diverse [3, 12]. Calcium is the most common cause of IOL opacification, especially in hydrophilic IOLs. Generally, calcium-related IOL opacification is irreversible, with a late onset over 3 months, and sometimes severe enough to impair visual performance [10, 13–17]. The risk factors of calcium-related IOL opacification are known to be diabetes mellitus, uveitis, postoperative inflammation, and intraocular calcium concentration [18–20]. However, considering the reversibility and short interval between IOL implantation and opacification in our cases, calcium deposit is not likely, though it cannot be completely excluded.

In our cases, the cloudy opacification started from peripheral optics and extended concentrically toward the center. Considering the clinical features of the connection between IOL opacities and anterior capsulorhexis, temporary growth of lens epithelial cell (LEC) cannot be completely excluded as a possible cause of IOL opacity. It is known that LECs can frequently grow out onto the IOL surface and can sometimes cause IOL decentration and capsular phimosis [21, 22]. Although, LEC outgrowth is known to be less severe on the hydrophobic acrylic IOL surface in comparison to hydrophilic IOLs, [4] one previous study revealed that LEC growth on hydrophobic acrylic IOLs was common and reached maximum at 30 days after surgery and then resolved spontaneously [21].

The early onset of opacification and its resolution with IOP control or topical steroid in our cases also suggests a possible relationship between the postoperative ocular environment and the opacification. Although a definite anterior chamber reaction was not observed in Case 1, a mild anterior chamber reaction was observed in Case 2. Even in Case 1, it is possible that some temporary inflammation existed and completely resolved before the follow-up visit and that IOL opacification was observed only in the quiet eye. In addition, instillation of Xalatan (latanoprost, prostaglandin analogue) might increase the vascular leakage in this case. Our group previously reported a similar, reversible, and bilateral IOL opacification of hydrophilic IOLs (Akreos MI-60, Baush & Lomb) in a diabetic patient who received bone marrow transplantation for chronic myelogenous leukemia [20]. In that case, the bilateral IOL opacification was completely resolved after intravitreal injection of anti-vascular endothelial growth factor, in effort to control cystoid macular edema [20].

There are previous reports of delayed-onset-type toxic anterior segment syndrome (TASS) with inflammatory plaque deposits on the IOL surface [23, 24]. Typical manifestations of TASS are acute onset (12–48 h after surgery), limbus-to-limbus corneal edema, and small amount of hypopyon. However, intense use of topical steroid in the early postoperative period can mask the typical TASS signs and can cause atypical and delayed manifestations, such as reversible IOL opacifications as in our cases. The resolution effect of potent topical steroid in Case 2 may also raise the possibility of TASS. Recently, an outbreak of subacute-onset of TASS related to hydrophobic acrylic IOLs was reported [25]. In that report, the time of onset of included 147 cases varied from 1 day to 88 days after surgery [25]. The common signs accompanied were accompanied by corneal edema, fibrinous inflammation and hypopyon [25]. However, a reversible IOL opacification was not reported. It seems that subacute or late onset TASS is not rare and may be related to various causes [23, 26, 27].

The limitation of this study is that the findings are mostly observational. Therefore, the materials and mechanisms of IOL opacification are still unknown. However, IOL explantation for laboratory test was not granted in both cases because IOL opacification disappeared with topical medications and patients’ visual complaints were completely resolved.

## Conclusions

In summary, we report two cases of completely reversible opacification of hydrophobic acrylic IOL (Tecnis ZCB00), observed within 2 months after surgery. Either temporary LEC outgrowth or delayed-atypical TASS can be the possibility.

## Abbreviations

CDVA: corrected distance visual acuity
IOL: intraocular lens
IOP: intraocular pressure
PCO: posterior capsule opacification
